# The Function behind the Relation between Lipid Metabolism and Vimentin on H9N2 Subtype AIV Replication

**DOI:** 10.3390/v14081814

**Published:** 2022-08-18

**Authors:** Anran Lu, Jing Yang, Xiangyu Huang, Xinmei Huang, Guihu Yin, Yiqin Cai, Xiuli Feng, Xiaofei Zhang, Yin Li, Qingtao Liu

**Affiliations:** 1 Key Laboratory of Veterinary Biological Engineering and Technology, Ministry of Agriculture, Institute of Veterinary Medicine, Jiangsu Academy of Agricultural Sciences, Nanjing 210014, China; 2 Key Laboratory of Animal Microbiology of China’s Ministry of Agriculture, College of Veterinary Medicine, Nanjing Agricultural University, Nanjing 210095, China; 3 Jiangsu Key Laboratory for Food Quality and Safety-State Key Laboratory Cultivation Base, Ministry of Science and Technology, Institute of Veterinary Medicine, Jiangsu Academy of Agricultural Sciences, Nanjing 210014, China

**Keywords:** H9N2 subtype AIV, cholesterol, HMGCR, AMPK, lipid rafts, vimentin

## Abstract

Avian influenza caused by H9N2 subtype avian influenza virus (AIV) poses a great threat to the healthy development of the poultry industry. Vimentin is closely related to intracellular lipid metabolism, which plays an important role during the viral infection process. However, the function of lipid metabolism and vimentin on H9N2 AIV replication is unclear. In this paper, the cholesterol level and 3-hydroxy-3-methylglutaryl coenzyme a reductase (HMGCR) phosphorylation were investigated in vimentin knockout (KO) and human cervical carcinoma cells (HeLa) cell with or without AIV infection. The results showed that compared to the control group without infected with H9N2 subtype AIV, the cholesterol contents were significantly increased, while HMGCR phosphorylation level was reduced in both KO and HeLa cell after virus infection. Furthermore, viral replication was significantly inhibited in the cells treated with the cholesterol inhibitor lovastatin. Compared with the control group, adenylate activated protein kinase (AMPK), a kinase regulating HMGCR enzymatic activity was inhibited in both KO and HeLa cells in the infected virus group, and AMPK phosphorylation levels were significantly lower in KO HeLa cell than that of HeLa cells. Additionally, after MβCD treatment, viral hemagglutinin (HA) gene level was significantly decreased in HeLa cells, while it was significantly increased in KO HeLa cells. In addition, vimentin expression was significantly increased in MβCD-treated HeLa cells with the viral infection and returned to normal levels after exogenous cholesterol to backfill the MβCD-treated cells. Therefore, the disruption of lipid rafts during the binding phase of viral invasion of cells significantly reduced viral infection. These studies indicated that the lipid rafts and cholesterol levels might be critical for H9N2 subtype AIV infection of human-derived cells and that vimentin might play an important role in the regulation of lipids on viral replication, which provided an important antiviral target against influenza virus.

## 1. Introduction

H9N2 avian influenza causes widespread transmission worldwide and is often accompanied by co-infection with other pathogens during infection in poultry, causing huge economic losses to the poultry industry [1,2]. H9N2 AIV has caused an endemic disease present in most parts of China since it was isolated from chickens in 1994. H9N2 AIV is a low pathogenic influenza virus, which is a single-stranded, negative-sense RNA virus with a capsid and a viral particle size of approximately 80–120 nm in diameter [3,4]. The infection mechanism of H9N2 subtype AIV has been the focus of scientific research.

As a member of the intermediate filament family, vimentin widely presents in the nucleus and organelles, as well as in the cytoplasm, and plays an important role in virus life cycle [5]. Vimentin is reported to promote the acidification of influenza virus endonucleosomes. The pH environment is essential for influenza virus infection, which triggers conformational changes in hemagglutinin and mediates the fusion of viral and endosomal membranes [6,7]. In H3N2 AIV infection, the impaired acidification of late intranuclear bodies in vimentin gene deficient cells leads to blocking the viral genome release, thereby significantly reducing viral RNA and protein expression, as well as the production of daughter viral particles and viral proliferation inhibition [8]. In addition, vimentin affects the transport of vRNPs and the activity of viral polymerase [9]. These results prove that vimentin plays the vital roles at different stages of AIV infection, and the mechanism of action on AIV still needs to be further explored.

The lipids on cell membranes are reported as co-receptors for viral invasion and facilitate viral entry into the cell. Non-enveloped viruses use sphingolipids on the host cell membrane as receptors and invade the host cell [10]. Viruses with capsid membranes require the assistance of low-density lipoprotein receptors (LDL-R) for successful invasion, such as hepatitis C virus (HCV) and bovine viral diarrhea-mucosal virus (BVDV) in the flaviviridae family [11,12]. During influenza A virus infection cycle, viral particles enter the host cell through the plasma membrane, in which lipids play an important role in viral infection into the host cells, and fatty acid, cholesterol, phospholipid and glycolipid metabolism participate in the viral infection processes [13]. Depletion of the host cell’s cholesterol and disruption of the lipid raft structure effectively reduce influenza A virus binding and internalization [14,15], and cholesterol is important for maintaining viral stability and infectivity [16]. Intact cellular and viral sphingolipids are essential for effective viral infection [17].

To investigate the effect of lipid metabolism and vimentin on H9N2 subtype AIV replication, in this paper, we investigated the effect of cholesterol, HMGCR, AMPK phosphorylation and lipid raft on viral replication in vimentin knockout (KO-vimentin) and HeLa cells. This study has important reference value for exploring the function and mechanism of vimentin and lipid metabolism on viral infection and provides an insight for antiviral targets development against AIV.

## 2. Materials and Methods

### 2.1. Virus and Cell

H9N2 AIV used in this study was isolated from the cloaca of a healthy chicken in Shandong 2017 [18]. HeLa cells (CCL-2) were maintained in Dulbecco modified Eagle medium (DMEM, 319-005-CL, Wisent, Nanjing, China) supplemented with 10% fetal bovine serum (FBS, 086-150, Wisent, Nanjing, China) and 5% CO_2_ at 37 °C.

### 2.2. Reagents and Antibodies

Vimentin monoclonal antibody was purchased from Abcam (Cambridge, UK). Influenza virus NP protein monoclonal antibody was made by our laboratory [19]. GAPDH monoclonal antibody was purchased from Enogene Biologicals (Nanjing, China). β-actin monoclonal antibody, AMPK monoclonal antibody and AMPK phosphorylated monoclonal antibody P-AMPK were purchased from BOSTER Biologicals (Nanjing, China). HMGCR monoclonal antibody and HMGCR phosphorylated monoclonal antibody P-HMGCR were purchased from Abmart Biologicals (Shanghai, China). HRP-labeled goat anti-rabbit/mouse IgG secondary antibody was purchased from Univ Biologicals (Shanghai, China). Lipid raft disruptor MβCD, water-soluble cholesterol and cholesterol inhibitor lovastatin were purchased from Sigma Aldrich (Burlington, MA, USA). Total cholesterol (TC) content assay kit and puromycin were purchased from Sangon Biotech (Shanghai, China).

### 2.3. Construction of Vimentin Knockout Cell Lines

Based on the vimentin gene on NCBI database (NM_003380.5), sgRNA are designed online (http//crispr.mit.edu accessed on 13 July 2022), shown in Table 1. The synthesized sgRNA oligonucleotide sequences were subjected to annealing reactions and ligated into the linearized PX459 vector. After sequencing identification, the recombinant vector was transfected into HeLa cells, which are culturing in DMEM medium with 10% FBS and 2.5 µg/mL puromycin. After four days, the screened transfected cells were cultured with DMEM medium only containing 10% FBS. The surviving mixed clones were verified, and HeLa cells with vimentin knockdown were named as KO HeLa cells.

### 2.4. Cholesterol Extraction and Assay

Cholesterol concentrations were determined using a colorimetric method following the instructions of total cholesterol extraction kit. Simply, the extraction solution was added at a ratio from 1:500 to 1:1000, and KO and HeLa cells were crushed using an ultrasonic crusher at 300 W, 2 S and 3 S intervals, for a total crushing time of 3 min. The 50 μmol/mL standard sample was diluted with the extraction solution into 2, 1.25, 0.625, 0.3125, 0.15625 and 0.078 μmol/mL, respectively. The test, standard and blank tubes were mixed thoroughly and detected according to the instructions. The absorbance value of each reaction sample at 500 Nanometer (nm) was measured using an enzyme standardizer, and the data were recorded and the standard curve was plotted according to the formula for calculating the cholesterol concentration.

### 2.5. CCK8 Assay for Cell Viability

KO and HeLa cells were treated with MβCD at 5, 8, 10 and 12 mM for 24 h, and cells without treatment were set as the control. Then, cells were incubated with cell counting kit-8 (CCK8) reagent (Sigma) for 1 h at 37 °C. The peak absorption of each well at 450 nm was then measured using an enzyme marker, and the data were analyzed.

### 2.6. MβCD Disrupts Cellular Lipid Rafts Assay

HeLa cells were treated in the following four ways: (1) treated with MβCD for 1 h to disrupt the rafts and then inoculated with virus; (2) inoculated with viral infection for 2 h and then treated with MβCD for 1 h; (3) pre-cooled at 4 °C for 30 min, treated with MβCD for 1 h and then inoculated with virus for 2 h at 4 °C; (4) pre-cooled at 4 °C for 30 min, inoculated with virus at 4 °C for 2 h and then incubated with MβCD at 37 °C for 1 h. All samples were collected at 24 h after viral infection to detect the effect of disruption of lipid rafts on virus replication of at the pre-infection, post-infection, virus binding to cell and fusion stages, respectively.

### 2.7. Exogenous Cholesterol Backfill Assay

After MβCD treatment for 1 h, KO and HeLa cells were incubated with exogenous water-soluble cholesterol for 1 h and incubated with virus at 37 °C for 2 h. Cell samples were collected to detect the viral gene levels according to different time points.

### 2.8. RNA Isolation and Quantitative Real-Time PCR

Total RNA was extracted from cells using Trizol (Takara, Japan), and RNA from each sample was reverse transcribed to cDNA using the PrimeScript RT Master Mix Kit (Takara, Japan). cDNA was isolated using TB Green Premix Ex Taq (TliRNaseH Plus) (Takara, Japan), and specific primers (Table 2) were used for quantitative real-time PCR (RT-qPCR) assays. Three sets of replicates were set up for each sample, the data were normalized to the β-actin expression level in each sample.

### 2.9. Western Blotting

The protein samples were collected from KO and HeLa cells and separated by SDS-PAGE, and electrotransferred onto the polyvinylidene difluoride (PVDF) membranes. After blocked, the electrotransferred PVDF membrane was incubated with the specific antibody overnight at 4 °C, and incubated with HRP-labeled secondary antibodies for 1 h at room temperature (RT). Protein levels were visualized with BIO-RAD Clarity Western ECL substrate.

### 2.10. Statistical Analysis

Replicated experimental samples were analyzed by *t*-test using Graphpad Prism 5.0 software to obtain the sample standard deviation (SD) of the mean. An * indicates the significant difference.

## 3. Results

### 3.1. Construction of Vimentin Knockout Cell Lines

**Figure 1:**
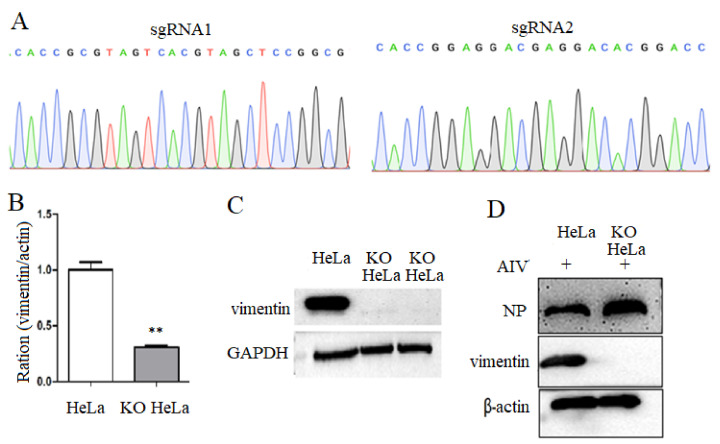
Construction and identification of vimentin gene knockout cell lines. (**A**) Sequencing analysis of recombinant vector. The designed sgRNA1 and 2 were inserted into PX459 vector and were identified with sequencing. (**B**) Vimentin mRNA level in KO cells. The recombinant vector was transfected into HeLa cells, and the vimentin gene level was knocked down. (**C**) Vimentin protein expression knockdown in KO HeLa cells. Vimentin protein expression was detected in KO HeLa cells. (**D**) NP protein expression increased in KO HeLa cells. After virus infection, viral NP protein expression was detected in KO HeLa cells with western blotting. ** *p* < 0.01.

To obtain the vimentin knockout cell lines, two sgRNA sequences were inserted in the recombinant plasmid, as shown in Figure 1A. After being transfected and screened with puromycin, it was found that compared with the HeLa cell controls, both mRNA level and protein expression of vimentin in the transfected HeLa cells were significantly decreased (Figure 1B,C), and vimentin knocked out cells were named KO-vimentin HeLa cells, abbreviated as KO HeLa cells. Additionally, it was observed that compared to that of HeLa cell control, NP protein expression was increased in KO-vimentin cells with viral infection (Figure 1D). The above results indicated that vimentin gene deletion cell line KO-vimentin has been successfully constructed.

### 3.2. H9N2 Subtype AIV Boosted the Intracellular Cholesterol Level

**Figure 2:**
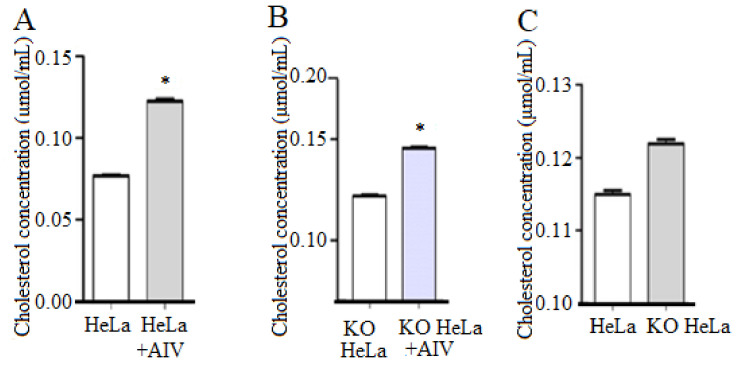
H9N2 subtype AIV infection increases intracellular cholesterol levels. (**A**) Total cholesterol levels of HeLa cells after infection. HeLa cells were incubated with H9N2 virus, and total cholesterol levels were detected. (**B**) Total cholesterol levels of KO HeLa cells after infection. KO HeLa cells were incubated with H9N2 virus, and total cholesterol levels were detected. (**C**) Total cholesterol levels of HeLa and KO HeLa cells. After viral infection, total cholesterol levels difference between HeLa and KO HeLa cells were detected. * *p* < 0.05.

To investigate the cholesterol level with AIV infection, KO and HeLa cells were treated with H9N2 AIV. It was found that compared with the non-infection control group, cholesterol in HeLa cells inoculated with H9N2 subtype AIV was significantly increased, indicating that the viral infection promoted cholesterol synthesis in HeLa cells (Figure 2A). Furthermore, the intracellular cholesterol levels in KO-vimentin cells infected with virus was higher than that of KO cells without infection (Figure 2B). Additionally, compared with HeLa cells, the cholesterol level in KO-vimentin cells was increased (Figure 2C). These results indicated that the intracellular cholesterol was increased after viral infection in both cell lines.

### 3.3. Activation of the Intracellular HMGCR by H9N2 Subtype AIV Infection

**Figure 3:**
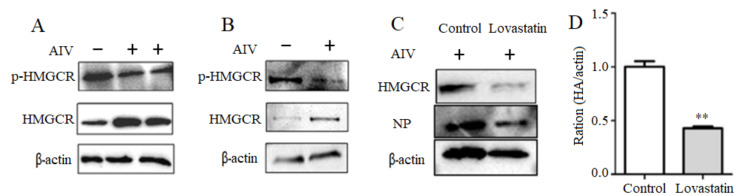
H9N2 subtype AIV infection activates HMGCR expression. HeLa and KO HeLa cells were inoculated with H9N2 virus, and the expression and phosphorylation of HMGCR in both cells were detected with western blotting. In addition, after HMGCR inhibitor Lovastatin treatment, viral NP protein expression and HA gene mRNA levels were detected. (**A**) The expression and phosphorylation of HMGCR protein on HeLa cells after viral infection. (**B**) The expression and phosphorylation of HMGCR protein on KO HeLa cells after viral infection. (**C**) NP protein in Lovastatin-treated cells after inoculation. (**D**) HA gene level in Lovastatin-treated cells after inoculation. ** *p* < 0.01.

To further understand the mechanism of cholesterol upregulation by H9N2 subtype AIV, the endogenous cholesterol rate-limiting enzyme HMGCR protein expression and phosphorylation level in cells infected with AIV were detected. Compared with the uninfected HeLa cell control, HMGCR protein expression was increased, and HMGCR phosphorylation in HeLa cells with viral infection was decreased (Figure 3A). Furthermore, the increased HMGCR protein expression and the decreased HMGCR phosphorylation were observed in KO HeLa cells (Figure 3B). These results suggested that H9N2 subtype AIV infection might attenuate the intracellular HMGCR phosphorylation and activate HMGCR protein expression, in which the function of vimentin on HMGCR protein expression and phosphorylation need to be explored.

To further understand the effect of HMGCR on the replication of H9N2 subtype AIV, it was observed that the expression of viral NP protein and intracellular HMGCR protein in HeLa cells treated with the HMGCR inhibitor Lovastatin were significantly reduced, compared with that of control group (Figure 3C). In addition, HA gene levels in Lovastatin-treated cells after infection were significantly decreased compared with those of the control (Figure 3D). These results indicated that Lovastatin could effectively inhibit the intracellular HMGCR activity and suppressed the viral proliferation.

The expression of viral NP protein and intracellular HMGCR protein in HeLa cell treated with were significantly reduced, compared with that of control group (Figure 3C). In addition, HA gene level in Lovastatin-treated cells after infection was significantly decreased compared with that of control without infection (Figure 3D).

### 3.4. H9N2 Subtype AIV Regulates HMGCR Enzyme Activity via AMPK

**Figure 4:**
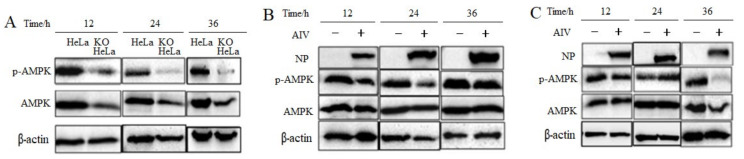
The cellular AMPK expression and phosphorylation level. AMPK protein expressions and phosphorylation in HeLa and KO HeLa cells with or without viral infection were detected with Western blotting. (**A**) The expression and phosphorylation of AMPK protein on KO HeLa cells compared with HeLa cells. (**B**) The expression and phosphorylation of AMPK protein on HeLa cells after viral infection. (**C**) The expression and phosphorylation of AMPK protein on KO HeLa cells after viral infection.

AMPK is reported to regulate HMGCR enzymatic activity [20]. To search the roles of viral infection on AMPK, KO and HeLa cells were incubated with H9N2 AIV. It was observed that AMPK expression and phosphorylation levels were significantly decreased in KO-vimentin cells, compared to HeLa cells (Figure 4A), indicating that vimentin deficiency might significantly inhibit the intracellular AMPK activity and thus affect the cholesterol levels. 

Furthermore, AMPK expression and phosphorylation levels in AIV-infected HeLa cells were examined by Western blotting. The results showed that the intracellular AMPK phosphorylation level was decreased (namely, AMPK inactivation) after viral infection at 12, 24 and 36 h, compared with the non-infected control group (Figure 4B). This indicated that intracellular AMPK was inhibited in H9N2 subtype AIV-infected cells, and the inhibited AMPK would down-regulate the HMGCR phosphorylation level and thus activate HMGCR activity.

To further investigate the function of vimentin on AMPK in KO-vimentin cells, AMPK level in KO-vimentin cells after viral infection was detected. The results showed that although no significant difference at 12 and 24 h, AMPK phosphorylation levels were significantly decreased at 36 after viral infection, compared with the non-infected control group (Figure 4C). The above results suggest that H9N2 subtype AIV infection of both cell lines might regulate the intracellular cholesterol levels mainly through the AMPK pathway.

### 3.5. Disruption of Lipid Raft Structure Affects H9N2 AIV Replication

**Figure 5:**
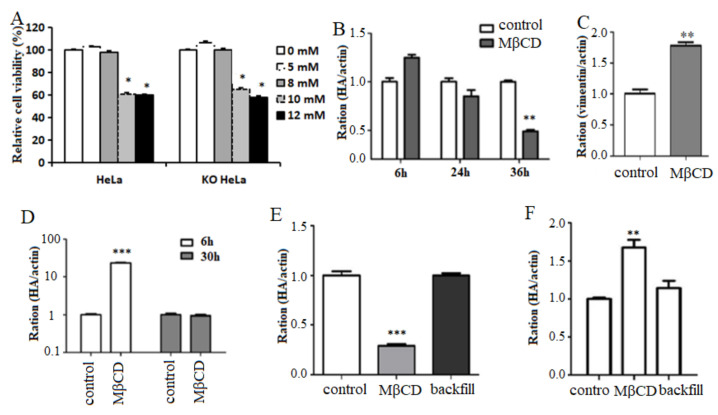
Disruption of lipid raft structure by MβCD affects the proliferation of H9N2 subtype AIV on cells. HeLa and KO HeLa cells were treated with MβCD to disrupt the lipid raft structure and incubated with H9N2 virus. The HA gene levels were detected with the fluorescence quantitative PCR. (**A**) The effect of MβCD on the viability of cells. (**B**) HA gene levels in MβCD-treated HeLa cells. (**C**) Vimentin gene levels in MβCD-treated HeLa cells. (**D**) HA gene levels in MβCD-treated KO HeLa cells. (**E**) Viral HA gene levels in HeLa cells after backfill assay. (**F**) Viral HA gene levels in KO HeLa cells after backfill assay. *** *p* < 0.001, ** *p* < 0.01, * *p* < 0.05.

Cholesterol was an important component of the lipid raft. Subsequently, the function of the lipid raft in viral replication was detected after disruption of lipid raft structure by MβCD treatment. The results showed that 8 mmol/L MβCD could effectively disrupt the lipid raft structure without affecting the cell activity in HeLa and KO HeLa cells (Figure 5A). The 8 mmol/L MβCD was selected as the experimental concentration for the following experiments. Compared with the control, HA gene level in HeLa cell with MβCD treatment was increased slightly at 6 h and decreased at 24 and 36 h after viral infection, and the decreasing level was significant at 36 h (Figure 5B), indicating that the replication of the virus might be inhibited in HeLa cells after the disruption of lipid rafts. 

Furthermore, vimentin gene levels were significantly increased in MβCD-treated HeLa cells compared to HeLa cells without MβCD treatment, indicating that the disruption of lipid raft structure in HeLa cell would have an effect on the intracellular vimentin gene levels (Figure 5C). In addition, the level of HA gene expression was increased significantly in MβCD-treated KO HeLa cells compared with the control group at 6 h of infection, and the difference in the viral HA level was not significant at 30 h after viral infection (Figure 5D), indicating that the disruption of the lipid raft structure in KO-vimentin HeLa cells promoted the replication of the virus in the early stage of infection in the cells.

After MβCD treatment, when the cells were incubated with exogenous water-soluble cholesterol for back-complementation (namely, backfill), it was found that in HeLa cells treated with MβCD, viral HA gene levels in the cholesterol back-complementation experimental group were restored to levels similar to those in the control without MβCD treatment (Figure 5E). In addition, the similar results were observed in KO HeLa cells with MβCD treatment and cholesterol back-complementation experimental group (Figure 5F). These results indicated that the disruption of lipid raft structure would affect H9N2 subtype AIV replication in host cells.

### 3.6. Role of Lipid Rafts in Cell Binding of H9N2 Subtype AIV

**Figure 6:**
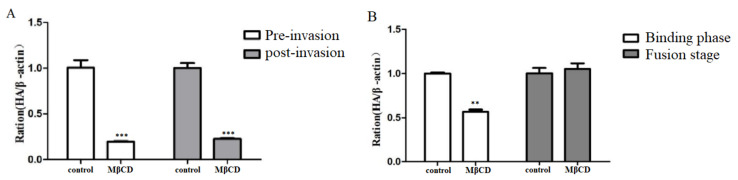
Role of lipid rafts in cell binding and fusion of H9N2 subtype AIV. HeLa cells were treated with MβCD at virus pre-invasion, post-invasion, binding and fusion stages. The viral HA gene levels were detected with fluorescence quantitative PCR. (**A**) Pre-invasion, post-invasion disruption of lipid raft structure inhibits virus proliferation. (**B**) Binding phase disrupts lipid rafts to inhibit viral proliferation. ** *p* < 0.01, *** *p* < 0.001.

To investigate the effect of lipid rafts on the viral infection stage, the viral HA gene levels at virus binding and fusion were detected. The results showed that disruption of lipid rafts with MβCD before and after viral infection significantly reduced the viral HA gene levels (Figure 6A). Furthermore, disruption of lipid rafts during the binding stage significantly reduced the viral HA gene levels during invasion (Figure 6B), indicating that lipid rafts might mainly affect virus–cell binding and have less effect on the fusion stage.

## 4. Discussion

Cytoplasmic intermediate filaments (cIFs) bind to the cellular lipids and play a role in the intracellular cholesterol transport [21,22]. Vimentin, a member of the intermediate filament protein family, is involved in the transport of intracellular lipoprotein-derived cholesterol [23,24], which is closely associated with the lipid metabolism in cells, especially cholesterol production. As an important component of lipid rafts, cholesterol plays an important role in viral invasion, replication and release [25,26]. As a key rate-limiting enzyme that regulates cholesterol synthesis in cells, HMGCR proteins intervene in the viral replication process, such as dengue virus (DENV) [27] and porcine circovirus (PCV) [28], Norwalk virus (NV) [29] and hepatitis C virus (HCV) [30]. However, the mechanism behind lipid metabolism and vimentin on H9N2 subtype AIV replication is unclear.

In this study, we first investigated the cholesterol level in H9N2 subtype AIV-infected HeLa and KO HeLa cells with vimentin gene deletion, and found that the cholesterol levels were significantly increased in both cell lines, in which cholesterol levels were found to be significantly increased in KO-vimentin cells compared to HeLa cells. In addition, HMGCR phosphorylation levels in both cell lines were reduced after the viral infection, indicating that H9N2 AIV-infected cells might increase the intracellular cholesterol content by activating HMGCR. This indicated that intracellular AMPK was inhibited in H9N2 subtype AIV-infected cells, and the inhibited AMPK would down-regulate HMGCR phosphorylation level and thus activated HMGCR activity. Vimentin deficiency significantly inhibited the intracellular AMPK activity and thus affected the cholesterol levels. It was reported that cholesterol-conjugated peptide and medicine zanamivir play inhibition on influenza viruses [31,32]. The above results suggest that H9N2 subtype AIV infection in both cell lines might regulate the intracellular cholesterol levels mainly through the AMPK pathway. Additionally, the viral replication was significantly inhibited in Lovastatin-treated cells, suggesting that the intracellular cholesterol content might be a crucial target for antiviral drug development against H9N2 AIV. 

The enzymatic activity of HMGCR was regulated by AMPK, and activation of AMPK upregulates the phosphorylation of HMGCR [20]. AMPK, a central regulator of cellular energy, is involved in the regulation of several cellular signaling pathways that affect the viral replication [33], including Epstein–Barr virus (EBV) [34], hepatitis C virus (HCV) [35] and dengue fever virus (DENV) [36,37]. In this paper, after infection with H9N2 subtype AIV, it was observed that in HeLa cells, AMPK phosphorylation level decreased significantly at 12 and 24 h. In addition, the phosphorylation level was significantly decreased at 36 h in KO-vimentin HeLa cells. Moreover, AMPK enzyme activity was significantly inhibited in KO-vimentin HeLa cells compared with control HeLa cells at experimental time points. These results suggested that the activity of HMGCR might be dominantly regulated by AMPK during H9N2 subtype AIV infection.

Lipid rafts are involved in various important biochemical processes in cells, such as signal transduction, cell migration and axon guidance [38,39,40]. Based on the above experimental results, to further investigate the role of lipid rafts in H9N2 subtype AIV-infected cells, MβCD was used to disturb the structural integrity of lipid rafts, and it was found that the viral HA gene expression level decreased significantly at 36 h of virus infection in HeLa cells and increased significantly at 6 h in KO-vimentin cells. It was also observed that vimentin levels were significantly increased in MβCD-treated HeLa cells, and using exogenous cholesterol to backfill the MβCD-treated group, the viral HA gene returned to normal levels compared to the control group.

Viruses with the vesicular membranes require lipid-mediated assistance for the successful host invasion. To further investigate the influence of lipid raft structure on the infection phase of H9N2 subtype AIV, both cell lines with the viral infection were performed using MβCD-treated cells. The results proved that the disruption of lipid rafts before invasion, after invasion and binding phase of invasion could significantly reduce the viral HA gene levels, while it was almost unaffected during the fusion phase. The results demonstrated the close link between vimentin protein, H9N2 subtype AIV replication and lipid metabolism.

## 5. Conclusions

**Figure 7:**
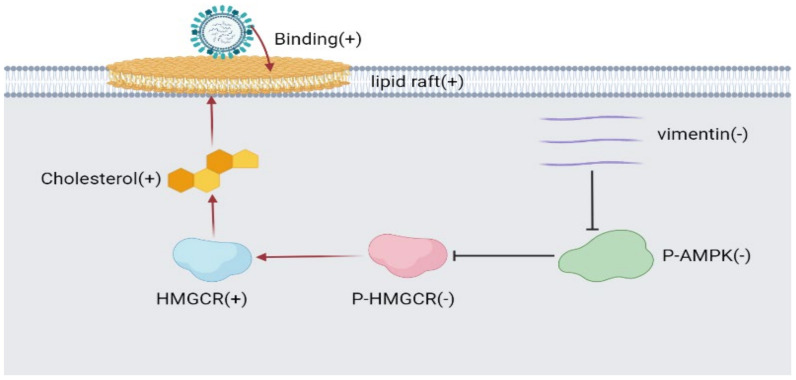
Model of lipid metabolism and vimentin on H9N2 subtype AIV. When vimentin was knocked down, the phosphorylation level of AMPK was decreased, resulting in the decreased level of HMGCR phosphorylation, which increased the enzyme activity, thereby increasing cholesterol. After the destruction of lipid rafts, the virus binding was inhibited, and cholesterol helped to stabilize the structure of lipid rafts, which might help the virus bind to cells.

There are various factors influencing the invasion of AIV into the host cells. In this paper, it was proven that the replication of H9N2 AIV on HeLa and KO-vimentin HeLa cell was associated with the cholesterol and cellular lipid rafts (Figure 7). In addition, H9N2 AIV upregulated the cholesterol levels by inhibiting AMPK activity and regulating HMGCR enzyme activity in both cell lines. Vimentin gene deletion also inhibited AMPK enzyme activity, leading to an increase in the the intracellular cholesterol levels. In further work, we will carry out research in the structural studies of AMPK and HMGCR on viral replication at the molecular level. This study suggested that vimentin might play an important role in the regulation of lipids on viral replication, which provide an important target against influenza viruses.

## Figures and Tables

**Table 1:** sgRNA sequences.

sgRNA	Oligonucleotide Sequences (5′-3′)
sgRNA 1S	CACCGCGTAGTCACGTAGCTCCGGC
sgRNA 1A	AAACGCCGGAGCTACGTGACTACGC
sgRNA 2S	CACCGGAGGACGAGGACACGGACC
sgRNA 2A	AAACGGTCCGTGTCCTCGTCCTCC

**Table 2:** Fluorescence quantitative PCR primer.

Primers	Sequences (5′→3′)	Amplification Size (bp)
β-actin	F: GCAAATTTCCATGGCACCGT R: GCCCCACTTGATTTTGGAGG	105
HA	F: TTACCCTGTTCAAGACGCCC R: GCCACACTCGTTGTTGTGTC	125
Vimentin	F: CACCAACGAGAAGGTGGAGC R: GACTTGCCTTGGCCCTTGAG	660

## Data Availability

Not applicable.
